# Design and biomechanical characteristics of porous meniscal implant structures using triply periodic minimal surfaces

**DOI:** 10.1186/s12967-019-1834-2

**Published:** 2019-03-18

**Authors:** Li-ya Zhu, Lan Li, Zong-an Li, Jian-ping Shi, Wen-lai Tang, Ji-quan Yang, Qing Jiang

**Affiliations:** 1 Jiangsu Key Laboratory of 3D Printing Equipment and Manufacturing, School of Electrical and Automation Engineering, Nanjing Normal University, Nanjing, China; 2 Nanjing Institute of Intelligent Advanced Equipment Industry Co., Ltd., Nanjing, China; 3 School of Mechanical Engineering, Southeast University, Nanjing, China; 4 State Key Laboratory of Pharmaceutical Biotechnology, Department of Sports Medicine and Adult Reconstructive Surgery, Drum Tower Hospital Affiliated to Medical School of Nanjing University, Nanjing, China

**Keywords:** Meniscal implant, Articular cartilage, Triply periodic minimal surface, Mechanical properties, Finite element analysis, 3D printing

## Abstract

**Background:**

Artificial meniscal implants can be used to replace a severely injured meniscus after meniscectomy and restore the normal functionality of a knee joint. The aim of this paper was to design porous meniscal implants and assess their biomechanical properties.

**Methods:**

Finite element simulations were conducted on eight different cases including intact healthy knees, knee joints with solid meniscal implants, and knee joints with meniscal implants with two types of triply periodic minimal surfaces. Compression stresses, shear stresses, and characteristics of stress concentrated areas were evaluated using an axial compressive load of 1150 N and an anterior load of 350 N.

**Results:**

Compared to the solid meniscal implant, the proposed porous meniscal implant produced lower levels of compression and shear stresses on the cartilage, which facilitated the cartilage to retain a semilunar characteristic similar to the natural meniscus. Moreover, both compression and shear stresses on the artificial cartilage were found to be sensitive to the pore properties of the meniscal implant. The meniscal implants with primitive surfaces (porosity: 41%) showed a better performance in disseminating stresses within the knee joint.

**Conclusion:**

The present commercial meniscal implant has the problem of equivalent biomechanical properties compared to natural menisci. The main advantage of the proposed porous structure is that it can be used to prevent excessive compression and shear stresses on the articular cartilages. This structure has advantages both in terms of mechanics and printability, which can be beneficial for future clinical applications.

## Background

The menisci are a pair of semilunar fibrocartilage structures between the load bearing joint surfaces of the femur and the tibia [1]. They play important roles in the knee including joint stabilization, mechanical shock absorption, load transmission, assistant lubrication, and nutrient supply to the articular cartilage [2]. The complex geometry, ultrastructure, and tissue composition of the menisci are responsible for their particular properties [3]. The geometry and position of the menisci are reported to be important because a meniscal extrusion can reduce the mechanical protection of the knee cartilage [4, 5]. Among two sides of the menisci, the lateral meniscus bears as much as 70% of the load in the lateral compartment and the medial meniscus carries approximately 50% of the medial load. The medial meniscus also restrains the anterior drawer as a block against the posterior part of the medial femoral condyle [6, 7]. Thus, it is vital to maintain an intact native structure of the menisci to ensure healthy functioning of the knee.

However, a deformity in the meniscus structure can result from certain diseases, degeneration, traumatic injuries, or abnormal development. In the adulthood, the vascularity of the meniscus is only limited to 10% to 30% of the peripheral zone [8]. The cells in the inner place are sparse and metabolize very slowly in getting oxygen and nutrition from the synovial fluid via diffusion. Hence, any tear in this region is very unlikely to heal by itself, leading to progressive joint degeneration and osteoarthritis [6, 9]. Surgical treatments commonly used to treat meniscal injuries are meniscectomy, sutures, and allograft transplantation.

Meniscectomy is the surgical removal of all or a part of an injured meniscus, classified as total meniscectomy or partial meniscectomy, respectively [10]. Meniscectomy may be beneficial in reducing the acute symptoms of a meniscus lesion [11]. Both total and partial meniscectomy have been shown to decrease the joint contact surface areas and increase the peak contact pressures. These can lead to radiographic degenerative changes, severe pain, and dysfunction, resulting in a decreased patient satisfaction [12, 13]. Moreover, approximately half of the patients who undergo meniscectomy develop symptomatic osteoarthritis. The removal of the menisci is now considered to have major detrimental consequences; thus, meniscal tissues should be retained whenever feasible. The primary advantage of sutures over meniscectomy is that a normal or near-normal meniscus can be retained if meniscal repair succeeded. However, current repair techniques are effective only in the outer periphery of the meniscus as this area is vascularized [14, 15]. In the innermost avascular zone of the meniscus, repair can hardly be done because of the less blood supply or less availability of the reparative cells [16].

Homologous meniscus (allografts) transplantation has been proposed as another possible solution for meniscal injuries. Studies have also proved that meniscal allograft transplantation can provide short or intermediate term improvements with regard to basic daily activities. However, meniscal allografts are known to shrivel and experience collagen remodeling upon transplantation, which can affect the mechanical strength and cause allograft tears, articular instability, and degenerative damages [17]. Other problems, such as limited donors, size mismatch and potential incompatibility, risk of disease transfer, rejection, infection, and high expenses, have also called for alternative treatment options [18–20].

A synthetic, non-resorbable total meniscus replacement can overcome the shortcomings associated with the use of meniscal allografts [21]. Such prosthesis needs to remain in the place and perform for many years. Thus, the challenge is to find a biomaterial that is capable of withstanding loading forces in the joint. Recently, anatomically-shaped polycarbonate urethane (PCU) meniscus implants have been developed and compared to the native meniscus. These implants have been demonstrated to be biocompatible and resistant to short-term physiological loading. However, they have been shown to significantly increase the peak pressure and reduce the contact area, resulting in a lack of improvement after meniscectomy [22]. One possible reason is that the elastic modulus of PCU is within the range of a natural cartilage but not the same as a natural meniscus [23]. According to the previous reports, implants with porous structures may be effective in eliminating this modulus mismatch and maintaining the material composition. Furthermore, a porous structure is beneficial for cell attachment and proliferation, which can offer a stable, long-term fixation via biological anchorage of the implant [24]. Thus, the aim of this study was to adjust the mechanical stiffness of a meniscal implant by developing an original solid prosthesis with porous structure.

The triply periodic minimal surfaces (TPMS) have served as a promising solution for porous microstructure design due to their intrinsic superior features, such as interconnectivity, tortuosity, and high surface to volume ratio. Recent studies on TPMS-based scaffolds have shown that these scaffolds can provide better mechanical and biological properties compared to the traditional scaffolds composed of rod connected porous structures [25, 26]. Such architecture reduces the stress concentrations that are seen in typical lattice network structures. Olivares et al. have indicated that a gyroid surface has higher capability in promoting the differentiation process compared to the conventional hexagonal architecture [27]. Abueidda et al. have investigated the electrical/thermal conductivities, elastic properties, and anisotropy using six TPMS units and found that a primitive unit has the highest shear modulus [28]. Furthermore, pore architectures of TPMS-based scaffolds can be rearranged from a macro-scale to a nano-scale according to the biological properties of the native tissues. In this paper, a modeling method was employed for designing porous meniscus implants with precisely controlled internal architectures and complex external anatomical shapes using TPMS. Then, the biomechanical characteristics of the implants were investigated. Stresses and their distributions in the knee joint were analyzed and compared using healthy intact knees, knee joints with solid meniscal implants, and knee joints with proposed porous implants via finite element analysis methods.

## Methods

In this study, all methods were carried out in accordance with the relevant guidelines and regulations. All experimental protocols were approved by the committee of Drum Tower Hospital affiliated to the Medical School of Nanjing University.

### Data acquisition

The magnetic resonance (MR) images and computed tomographic (CT) images were obtained from a 35-year-old male participant with a healthy right knee joint. The MR scan was performed using a 3-T clinical MR scanner (uMR 770, United Imaging, Shanghai, P.R.C). The CT scan was performed using GE Lightspeed 16 CT equipment (GE, CT, USA). The MR images were obtained at intervals of 1.5 mm in the sagittal plane. The acquisition parameters were a field of view (FOV) of 240 × 228 mm and a resolution of 0.67 × 0.63 × 0.64 mm^3^ pixels. The CT images were obtained at intervals of 0.625 mm with a FOV of 500 mm.

### Finite element modeling and material properties

**Fig. 1:**
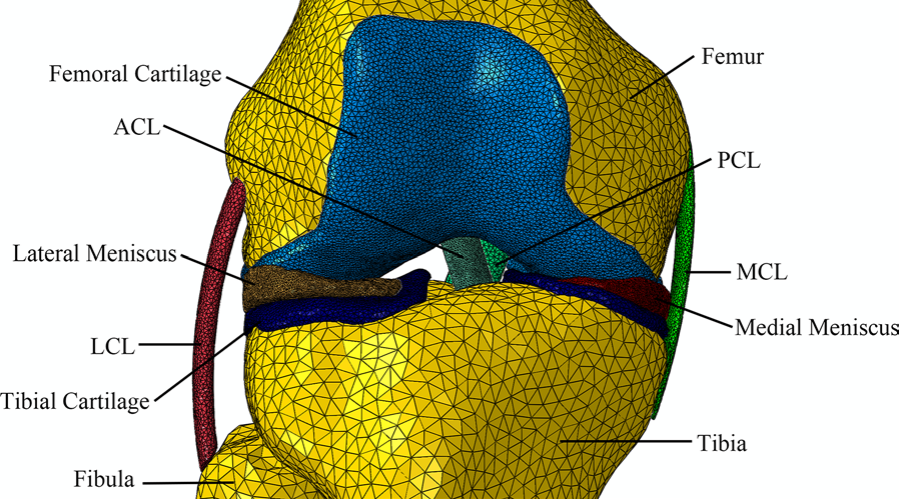
3D finite element model of the human knee joint (posterior view), including the bones, the articular cartilages, the ligaments and the menisci

The DICOM image files were imported to the software MIMICS 19.0 (Materialise, Leuven, Belgium) and segmented on the basis of gray intensities. The models of separate bone structures were accomplished with CT Bone Segmentation operation. The models of articular cartilages (including femoral, tibial, and patellar), menisci (including medial and lateral) and ligaments [including medial collateral (MCL) and lateral collateral (LCL)], anterior cruciate (ACL) and posterior cruciate (PCL), and patellar tendon were segmented from the MR images. All reconstructed models were exported as stereolithography (STL) files and re-meshed with Materialise 3-matic 11.0 software (Materialise, Leuven, Belgium). Then, the models were imported and assembled in Abaqus 2017 (SIMULIA, Rhode Island, USA). A finite element model of the knee joint was developed (Fig. 1). The ligaments, as nonlinear materials, were meshed with 10-noded quadratic tetrahedron (C3D10H). Other tissues, considered as linear materials, were meshed using 4-noded liner tetrahedron (C3D4).

The articular cartilages and menisci were assumed to be linear, elastic and isotropic with an elastic modulus (*E*) of 15 MPa and a Poisson ratio (*v*) of 0.475 [29–31]. The menisci were also modeled as linear, elastic and isotropic with parameters as follows: *E* = 120 MPa and *v* = 0.45. The bony tissues (femur and tibia) were considered as linear materials with an elastic modulus (*E*) of 7300 MPa and a Poisson’s ratio (*ν*) of 0.3 [32].

The ligaments were modeled as transversely isotropic nearly-incompressible neo-Hookean materials with a strain-energy function [33, 34]:

1 $$\varPhi = C_{10} \left( {\bar{G}_{1} - 3} \right) + \frac{1}{{D_{1} }}\left( {J_{F} - 1} \right)^{2} + S\left( \lambda \right)$$

Here, *S* (*λ*) denotes the strain energy function of the fiber family that satisfies the conditions:

2 $$\lambda \frac{ds}{d\lambda } = \left\{ {\begin{array}{ll} {0,}\hfill &\quad{\lambda \le 1} \\ {C_{3} \left( {e^{{\left( {\lambda - 1} \right)C_{4} }} - 1} \right),}\hfill &\quad{1 < \lambda < \lambda^{*} } \\ {C_{5} \lambda + C_{6} ,}\hfill & \quad{\lambda \ge \lambda^{*} } \\ \end{array} } \right.$$

where, *C*_10_ is the bulk material constant related to the shear modulus *μ* (*C*_10_ = 2/*μ*); *J*_*F*_ is the Jacobian of the deformation gradient **F**; and $\bar{G}_{1}$ represents the first invariant of the left Cauchy-Green tensor $\bar{G}_{1} = tr\overline{{{\mathbf{FF}}}}^{\text{T}}$ with the modified deformation gradient ${\bar{\mathbf{F}}}\left( {{\bar{\mathbf{F}}} = J_{F}^{ - 0.33} {\mathbf{F}}} \right)$.

The fiber stretch, *λ*, denotes the stress in the fibers, which is determined from the deformed fiber orientation ***a***_**d**_, the deformation gradient ***F***, and the initial fiber orientation ***a***_**0**_ (*λ* ·*** a***_**d**_ = ***F*** · ***a***_**0**_). The stiffness of these fibers increases exponentially when the fiber stretch is between one and a pre-defined value (λ*). When under compression (*λ *≤ 1), the fibers cannot support any compressive stress. When stretched beyond the pre-defined value, the fibers can get straighten and the stiffness can increase linearly. The initial fiber orientation was in line to the principle axis of the geometry. The constant *C*_3_ scaled the exponential stress, *C*_4_ was related to the rate of collagen uncramping, and *C*_5_ represented the elastic modulus of the straightened collagen fibers. The constant *C*_6_ was introduced to ensure stress continuation at $\lambda^{*} \left[ {C_{6} = \left( {e^{{C_{4} \left( {\lambda^{*} - 1} \right)}} - 1} \right) \cdot C_{3} - \left( {C_{5} \lambda^{*} } \right)} \right]$. The material constants *C*_10_, *C*_3_, *C*_4_, *C*_5_, and *D*_1_, are listed in Table 1.

**Table 1:** Material constants for the ligaments

	*C*_10_ (MPa)	*C*_3_ (MPa)	*C*_4_ (–)	*C*_5_ (MPa)	*D*_1_ (MPa^−1^)	λ* (–)
ACL	1.95	0.0139	116.22	535.039	0.00,683	1.046
PCL	3.25	0.1196	87.178	431.063	0.0041	1.035
LCL	1.44	0.57	48.0	467.1	0.00126	1.063
MCL	1.44	0.57	48.0	467.1	0.00126	1.063
PT	3.25	0.1196	87.178	431.063	0.0041	1.035

### Boundary and loading conditions

Slight flexion (4° flexion angle) simulation of the knee joint was considered in this study. In all models, the bottom of the tibia and the fibula was fixed in all translational and rotational degrees of freedom, whereas the femur was vertically constrained in the axial direction; all other rotational directions were left unconstrained. All ligaments were rigidly attached to their corresponding bones to simulate the bone-ligament attachment. For both lateral and medial hemi joints, distributing coupling constraints were modeled between the femur and meniscus, meniscus and tibia, and femur and tibia. Then, an axial compressive load of 1150 N and an anterior load of 350 N (60% of the body weight) were separately applied on the top and the anterior side of the femur.

### Porous meniscal implant design based on TPMS architectures

**Fig. 2:**
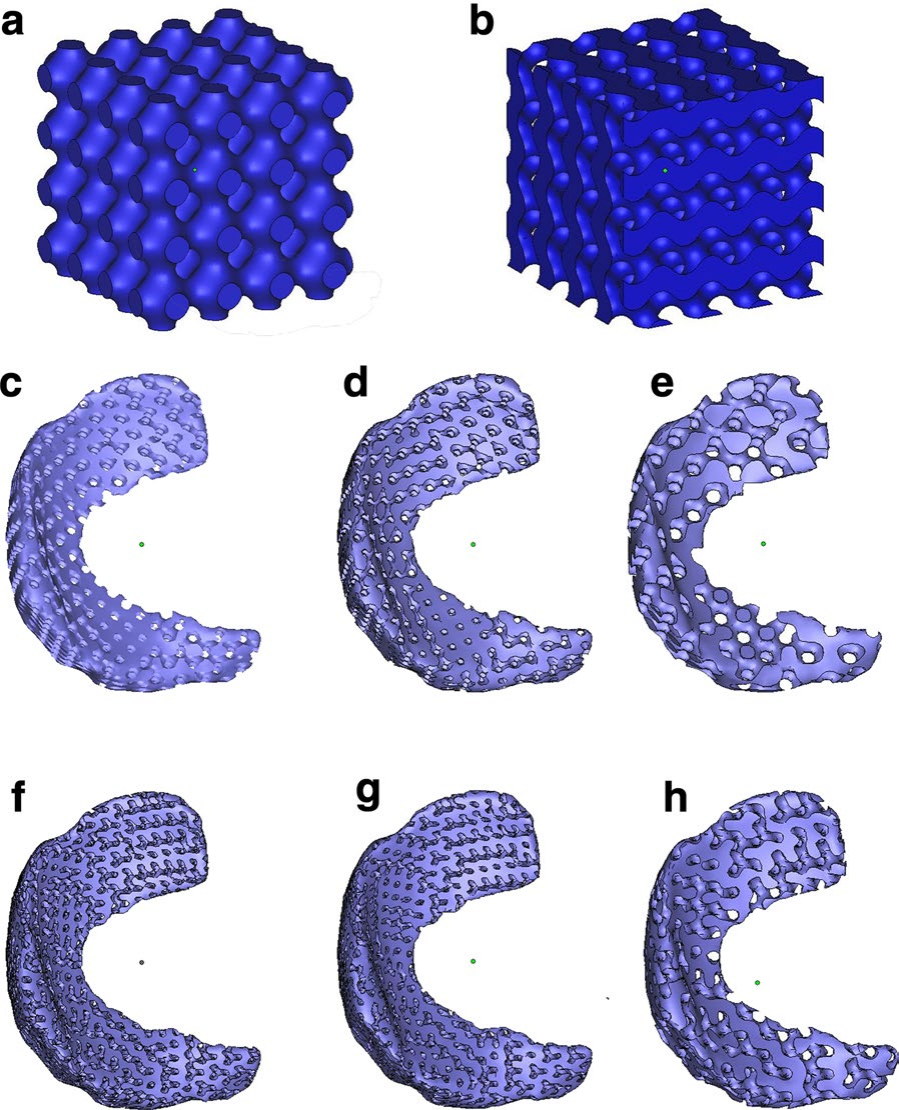
Porous meniscal implant modeling based on TPMS surfaces: **a** primitive surface, **b** gyroid surface, **c** porous implant structure based on P surface with 47% porosity, **d** porous implant structure based on P surface with 41% porosity, **e** porous implant structure based on P surface with 47% porosity, **f** porous implant structure based on G surface with 45% porosity, **g** porous implant structure based on G surface with 37% porosity, **h** porous implant structure based on G surface with 47% porosity

Two basic porous TPMS structures, known as primitive and gyroid surfaces, were considered in this study (Fig. 2a, b). Based on the previous research [25, 35], these porous structures can be represented by the following functions:

Primitive (type P) TPMS:

3 $$\phi_{\text{P}} (x,y,z) = \cos (ax) + \cos (by) + \cos (cz) + d$$

Gyroid (type G) TPMS:

4 $$\phi_{\text{G}} (x,y,z) = \cos (ax)\sin (by) + \cos (by)\sin (cz) + \cos (cz)\sin (ax) + d$$

The pore size and surface architectures are controlled by the parameters *a*, *b,* and *c* in the above functions. The porosity is controlled by the parameter *d* [36]. An area of *Φ *≥ 0 represents the solids and *Φ *< 0 represents the pores.

The Primitive and Gyroid TPMS units were then combined with a given cylinder external shape for further implant modeling [37]. The modes were defined by Boolean set expression as follows:

5 $$\begin{aligned} & S_{1} \cap S_{2} \hfill \\ & S_{1} = \left( {\phi_{\text{ex}} \le 0} \right) \hfill \\ & S_{2} = \left( {\phi_{\text{P}} > 0} \right)\;\;or\;\;S_{2} = \left( {\phi_{\text{G}} > 0} \right) \hfill \\ & \phi_{\text{ex}} = x^{2} + y^{2} - r^{2} \hfill \\ \end{aligned}$$

where, *r* is the radii of the cylinder. The proposed mathematical methods were performed using Wolfram Mathematica 11.0 software (Wolfram, Champaign, USA). Inequalities used in Eqs. (3)–(5) were used as the input and the “RegionPlot3D” command was used to generate all figures. Then, the “Export” command was used to save the models as STL files. Then, we attempted to construct a variety of TPMS-based porous scaffolds with similar anatomical shape of a meniscus (Fig. 2c–h). Boolean operations were completed using Materialise magics 20.03 software (Materialise, Leuven, Belgium).

## Results

**Fig. 3:**
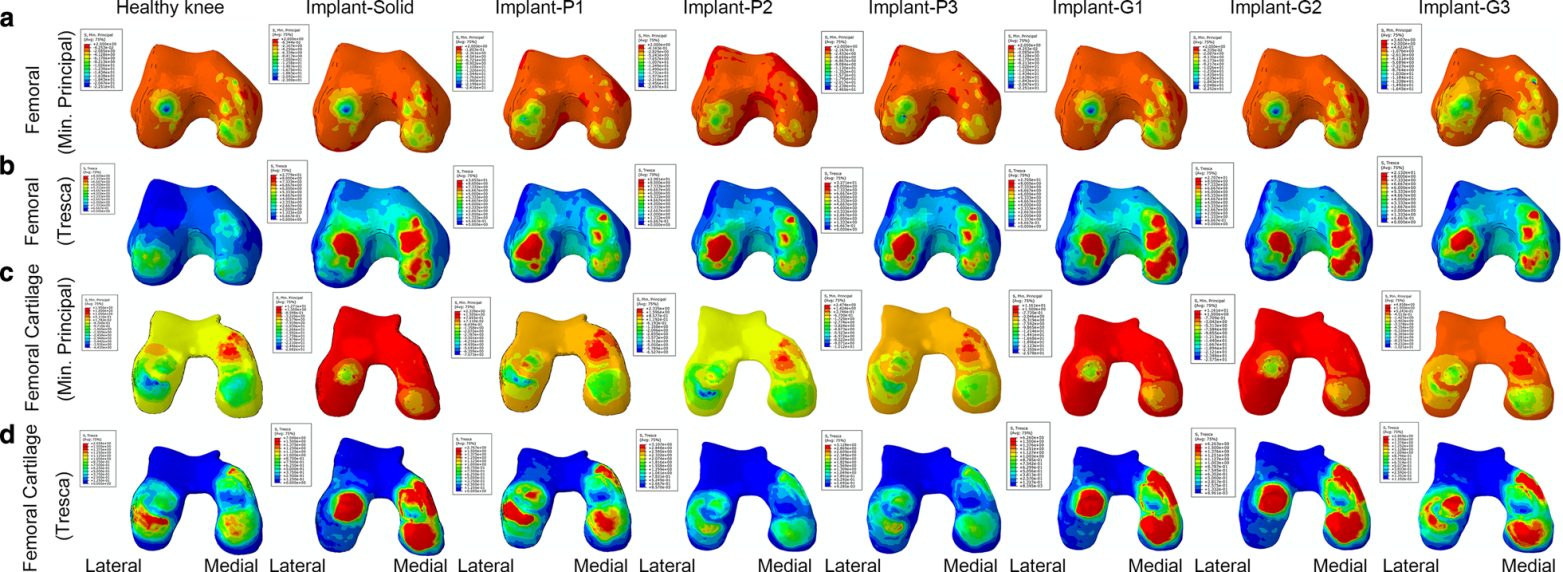
**a**–**d** The compression stress (minimum principal) and shear stress on femoral and femoral cartilage for the healthy knee, the knee joint with solid meniscal implant and with porous meniscal implants based on P and G surfaces

**Fig. 4:**
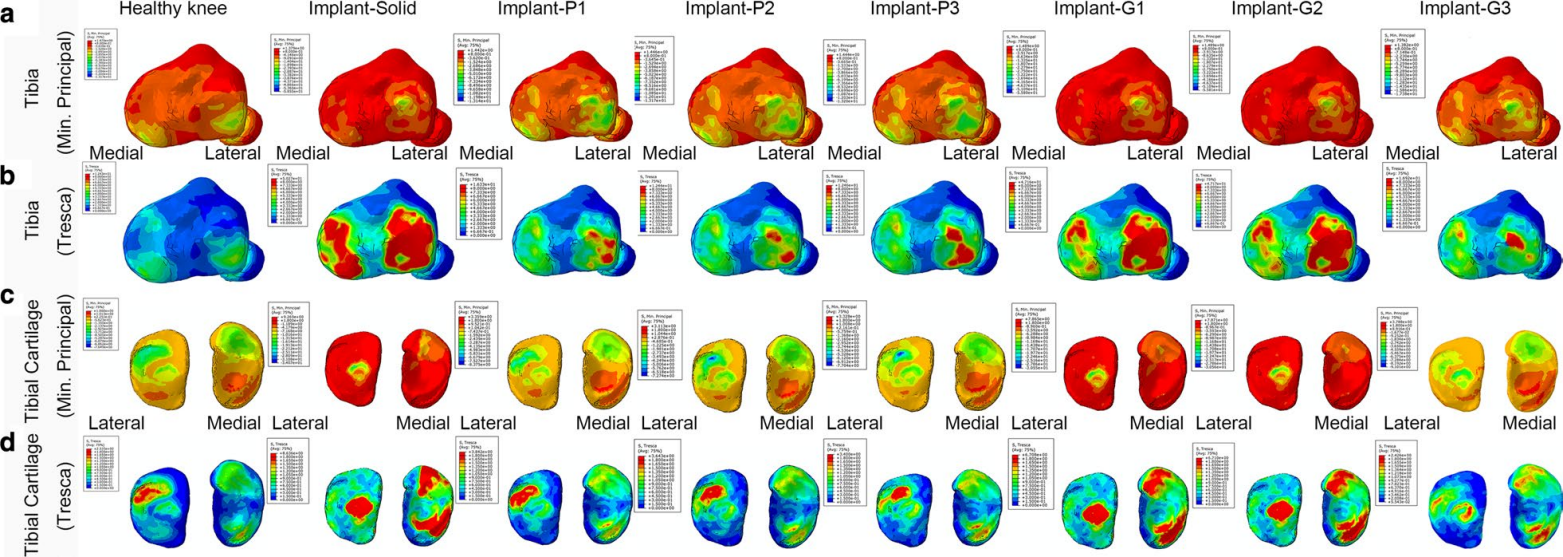
**a**–**d** The compression stress (minimum principal) and shear stress on tibia and tibial cartilage for the healthy knee, the knee joint with solid meniscal implant and with porous meniscal implants based on P and G surfaces

The results of finite element analysis obtained from the intact healthy knee were compared to the knee joints with either solid meniscal implants or TPMS-based porous meniscal implants. As seen in Figs. 3 and 4, both compression and shear stresses were disseminated over the femoral and tibial cartilages within the knee joint. However, a change in geometry of the lateral meniscus can cause a redistribution of stresses on both sides of the articular cartilages in the knee joint. Concentrated compression and shear stresses produced on the cartilage-to-meniscus contact areas of the femoral and tibial cartilages can be reduced by taking advantage of the porous meniscal implants instead of solid implants.

**Fig. 5:**
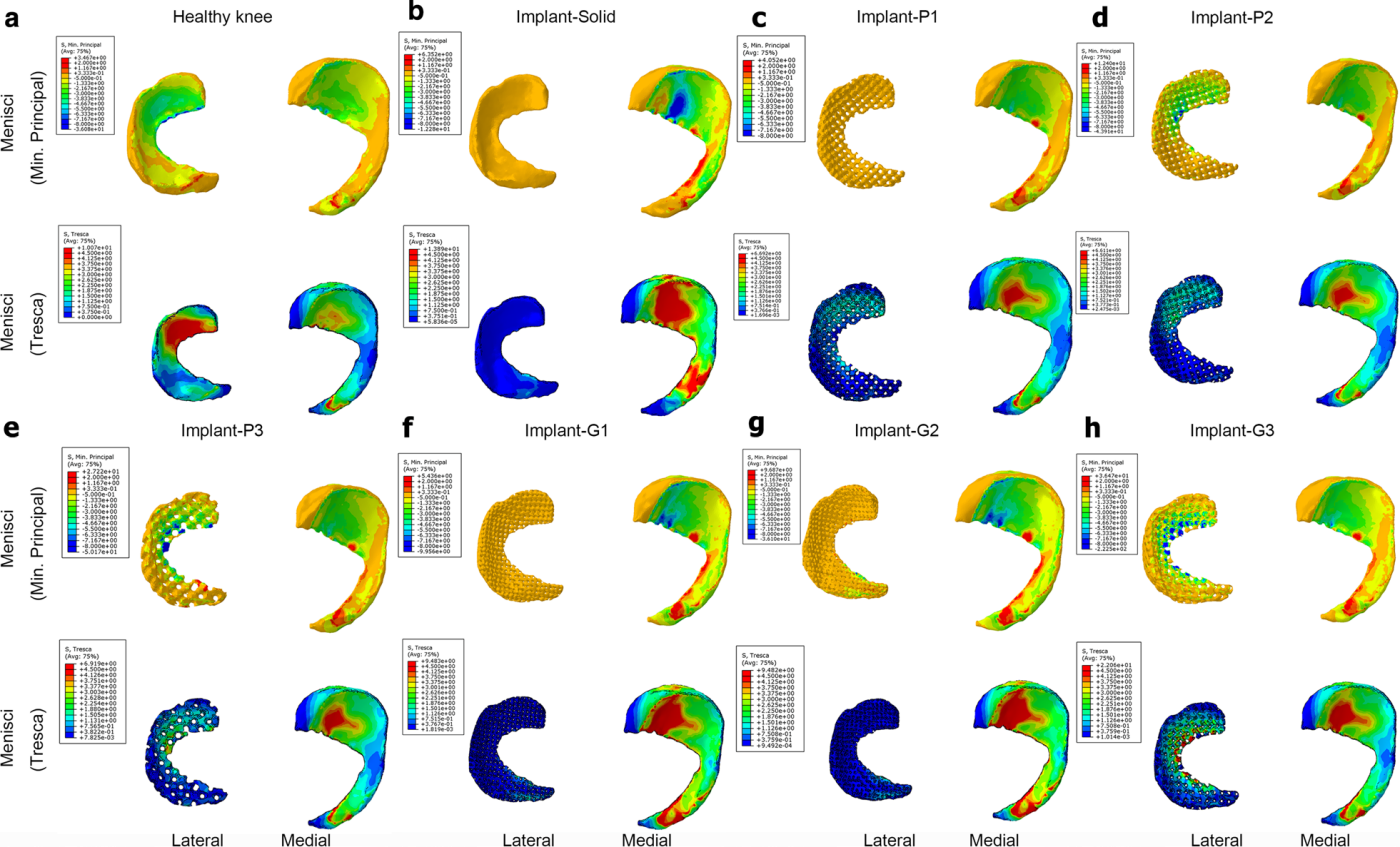
**a**–**h** The compression stress and shear stress on the menisci for the healthy knee, the knee joint with solid meniscal implant and with porous meniscal implants based on P and G surfaces

Figure 5 shows the compression and shear pressure distribution in the menisci under different types of medial meniscus. The area of concentrated compression and shear stresses in the lateral meniscus was mostly reduced in case of the knee joint with solid implant compared to that of the healthy knee. However, in the medial meniscus, this area was mostly broadened. This particular trend reversed completely with the use of various porous implants. It was also observed that a larger stress area in the lateral meniscus reduced the stress level for both femoral and tibial cartilages.

**Fig. 6:**
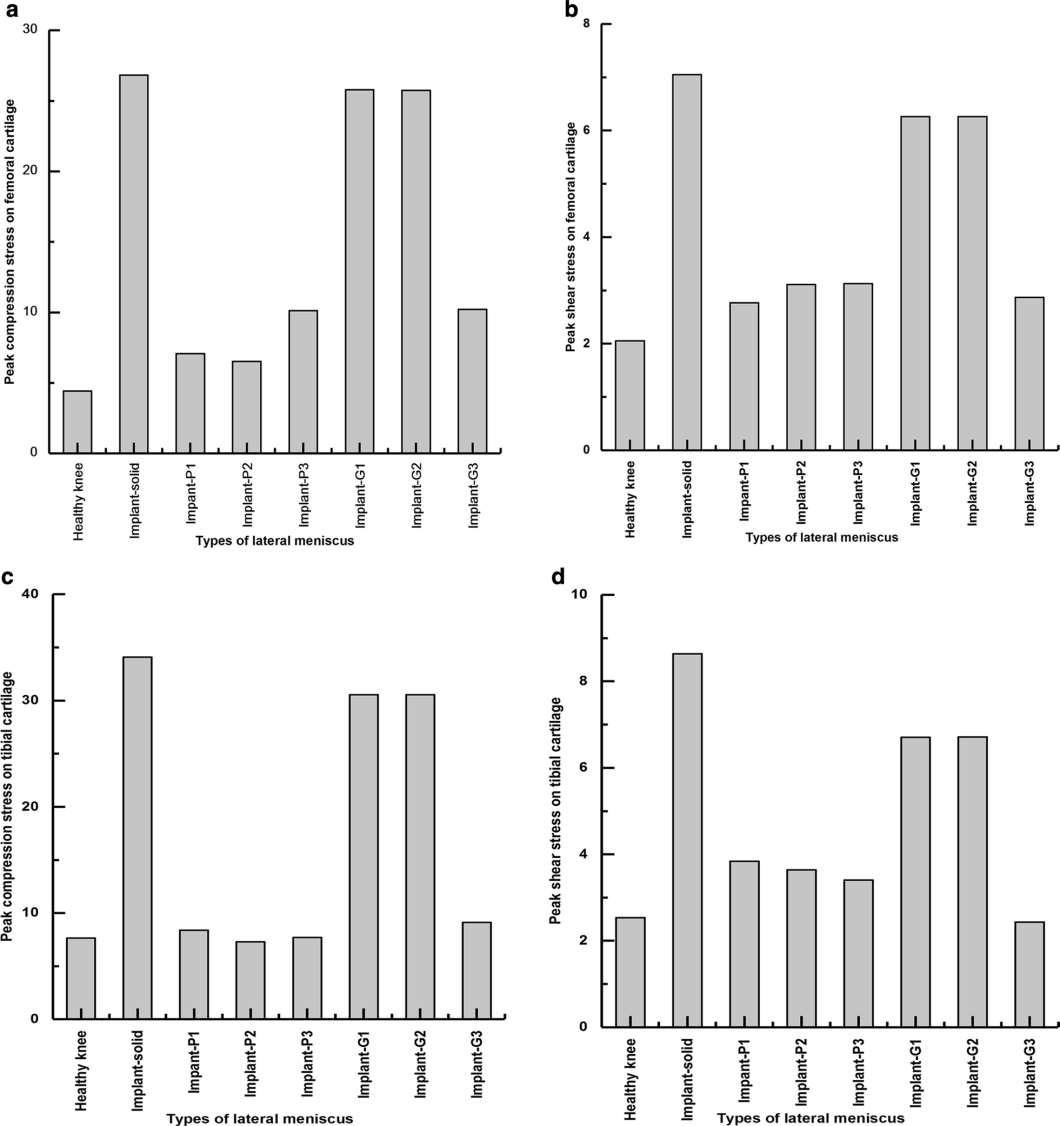
Peak value of compression stress and shear stress on the articular cartilages of knee joint in each case: healthy knee, knee joint with solid meniscal implant and with porous meniscal implants based on P and G surfaces

The peak compression and shear stress values are plotted in Fig. 6. The maximum compression stress produced on the articular cartilages by the knee joint with solid implant (femoral cartilage: 26.8 MPa; tibial cartilage: 34.1 MPa) was considerably higher than that by the healthy knee (femoral cartilage: 4.4 MPa; tibial cartilage: 7.6 MPa) as well as the knee joints with porous meniscal implants (Fig. 6a, c). Among all proposed porous meniscal implants, the knee joint with meniscal implant-P2 produced the lowest compression stress on both femoral and tibial cartilages (femoral cartilage: 6.5 MPa; tibial cartilage: 7.3 MPa). In addition, the maximum compression stress produced by the knee joint with G-surface-based meniscal implant was relatively higher than that by the knee joint with P-surface-based meniscal implant (average value of maximum compression stress on the femoral cartilage: 20.6 MPa vs 7.9 MPa; tibial cartilage: 23.4 MPa vs 7.8 MPa).

The maximum shear stress produced on the articular cartilages by the knee joint with solid implant (femoral cartilage: 7 MPa; tibial cartilage: 8.6 MPa) was also higher than that by the healthy knee (femoral cartilage: 2.1 MPa; tibial cartilage: 2.5 MPa) as well as the knee joints with porous meniscal implants (Fig. 6b, d). Among all proposed porous meniscal implants, the knee joint with meniscal implant-P1 produced the lowest shear stress on the femoral cartilage (2.8 MPa), whereas the knee joint with implant-G3 generated the lowest shear stress on the tibial cartilage (2.4 MPa).

## Discussion

The aim of this study was to estimate the influences of different structural properties of meniscal implants on the mechanical response of articular cartilages of a knee joint loaded with typical forces. Substituting an injured meniscus with an anatomically-shaped artificial implant has become a promising treatment for patients suffering from meniscal degeneration, diseases, or injuries. It has been reported that the mechanical properties of PCU are similar to that of the cartilages. However, the synthetic total meniscal implant designed in this study was found to greatly increase the peak pressure and reduce the contact area. For meniscal implants, an improvement in stiffness can play a crucial role in improving pressures experienced by the knee joint.

Taking advantages of medical imaging and advanced computing technologies, an anatomically accurate 3D finite element model of a knee joint was designed. The results of finite element analysis showed that the peak cartilage compression and shear pressures produced by the knee joint with solid implant were much higher than that by the healthy knee. Both compression and shear stresses produced on the femoral and tibial cartilages by this implant were concentrated in the central region and did not retain the semilunar characteristics of a natural meniscus. The solid nature of the commercial implants might not adequately restore the normal mechanics of a joint.

The most important finding of the current study was that compared to the solid meniscal implant, the proposed total meniscus implant significantly reduced both compression and shear stresses. In addition, a variation in porous structure of the meniscal implant affected both compression and shear stresses produced on the articular cartilage. In comparison to gyroid structures, primitive structures showed lower compression stresses. Since stresses are concentrated only at the connections between basis atoms [38], the spherical shape of primitive structures can facilitate an uniform and efficient transfer of the load from the top to the bottom. When a compressive load is applied to primitive structures, a compressive stress is generated only at the connections that are parallel to the loading direction. In contrast, when a load is applied to gyroid structures, both compressive and shear stresses are always generated at the connections regardless of the loading direction. However, a stress is generated at all the connections when a shear load is applied. In comparison to primitive structures, gyroid structures appear to be relatively weaker under compressive loading than that under shear loading. This may be because of the absence of connections parallel to the loading direction. The connections in primitive structures become relatively strong under compressive loading compared to that under shear loading.

On the other hand, it can be concluded that surfaces with higher curvature have more local stress distribution due to division of the force component into several components with different magnitudes and directions. Highly collapsed peanut-like shapes with large positive and negative curvatures acquired by gyroid structures can act as stress accumulators and are associated with a higher local stress concentration. In this study, the porous implant structure P2 (with 41% porosity) was capable of bearing greater compression stress (0.08) in comparison to implants P1 and P3 (with 47% porosity). Therefore, owing to the primitive surface, P2 structure with higher density can absorb more energy and bear more compression stress in the elastic area. However, in implant structures with gyroid surfaces, the peak value of shear stress was more susceptible to the size of pore architecture rather than porosity. Implant structure G3 (with pore size 700 μm) revealed much lower shear stress (0.21) compared to that of implants G1 and G2 (with pore size 500 μm). Considering the peak value of compression and shear stresses as well as the stress concentrated area, the knee joint with meniscal implant-P2 might be the most suitable choice.

**Fig. 7:**
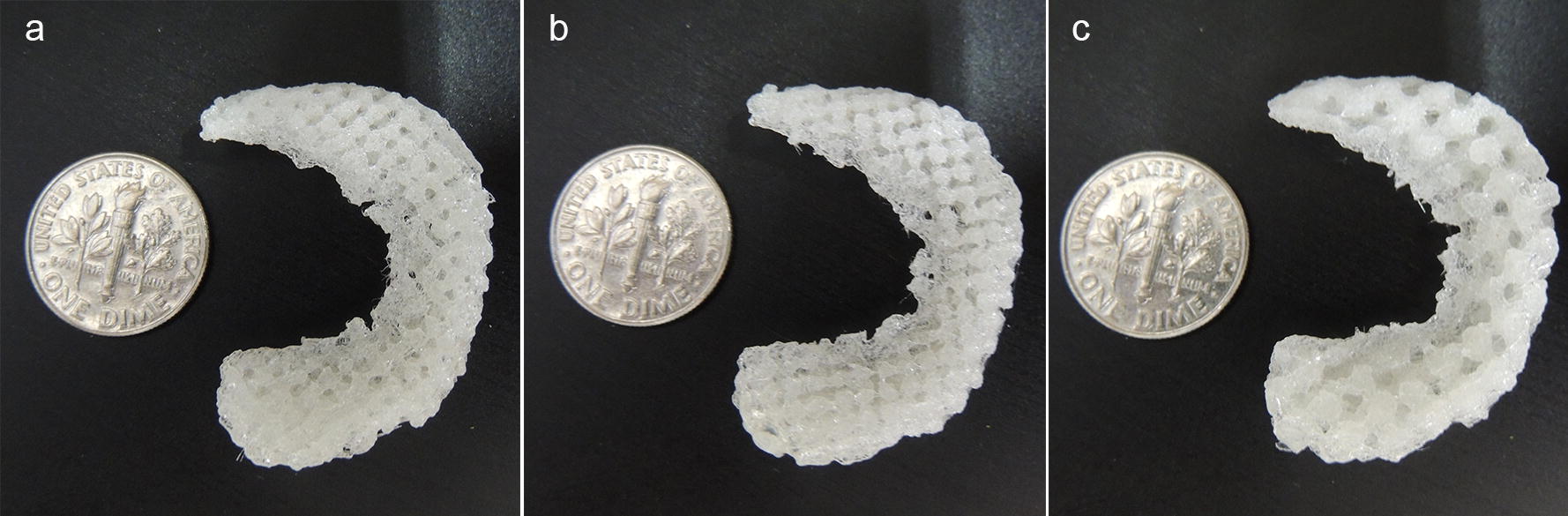
Photos of 3D printed P surface-based meniscal implant: **a** with 47% porosity, **b** with 41% porosity, **c** with 47% porosity

Additive manufacturing, such as fused deposition modeling (FDM), is an advanced manufacturing technique capable of fabricating custom parts with controlled internal structures [39]. Thus, this study investigated the potential of using FDM 3D printing for fabricating the proposed primitive-surface-based porous artificial meniscus made of PCU material (Fig. 7). However, it is challenging to overcome the inherent surface roughness of 3D printed surfaces. Improvements can be made in FDM technology and surface treatments can also be considered to make the surface smoother.

Some limitations should be addressed when interpreting the results of this study. Firstly, the finite element model was built using the geometric information from a single subject data. Thus, the outcomes related to loading conditions applied in this study may differ with another model. Secondly, standard static simulations were carried out in the knee with simple loading and boundary conditions. The articular cartilages and menisci were assumed to be linear, elastic and isotropic. The viscoelastic and swelling material properties were not considered while modeling the menisci and the articular cartilage. In future, dynamic finite element simulations should be performed with complete gait cycle loading and boundary conditions to better predict the biomechanics of a knee joint. Thirdly, although PCU is known to be softer at body temperature and in a fully humidified environment, the properties of all materials considered in this study were evaluated at room temperature [40]. Thus, the influence of temperature on PCU material and the inner environment of the knee joint should be considered in further finite element simulations. Lastly, the experiments were carried out using a limited number of meniscal implants. Thus, the biomechanical effects of variations in pore size, porosity, and pore shape of the meniscal implant should also be studied in future.

## Conclusion

In this study, an anatomically-shaped porous meniscal implant was proposed using TPMS structures. Finite element simulations were conducted to assess and compare the biomechanical properties of the proposed implant with the intact healthy knee and the knee joint with solid meniscal implant. Compared to the solid meniscal implant, the anatomically-shaped porous meniscal implants can prevent higher magnitude compression and shear stresses on the articular cartilage. Concentrated compression and shear stresses produced at the cartilage-to-meniscus contact areas of the artificial cartilages can be controlled and a semilunar characteristic similar to the natural meniscus can be retained. Furthermore, the proposed porous meniscal implants can be fabricated with FDM technology, which can have a great clinical application value.

## Abbreviations

PCU: polycarbonate-urethane
TPMS: triply periodic minimal surfaces
FOV: field of view
FE: finite element
FDM: fused deposition modeling
ACL: anterior cruciate ligament
PCL: posterior cruciate ligament
LCL: lateral collateral ligament
MCL: medial collateral ligament
PT: patellar tendon
